# A rare case of dengue encephalopathy complicating a term pregnancy

**DOI:** 10.1186/s13104-017-2391-5

**Published:** 2017-02-02

**Authors:** Lavanya Rajagopala, Ravindra L. Satharasinghe, Madhava Karunarathna

**Affiliations:** Sri Jayewardenepura General Hospital, Sri Jayewardenepura, Kotte, Sri Lanka

**Keywords:** Dengue fever, Encephalopathy, Term pregnancy

## Abstract

**Background:**

Dengue fever has an expanded clinical spectrum ranging from an asymptomatic infection to life threatening dengue hemorrhagic fever and refractory shock. Dengue infection in pregnancy can be a diagnostic dilemma, particularly considering the physiological changes in pregnancy and the obstetric complications encountered in clinical practice. Hence the knowledge of its diagnosis and management in its atypical presentations is of paramount importance. Here we report an unusual case of uncomplicated dengue encephalopathy in a term mother, probably the first to be reported from the Indian subcontinent.

**Case Presentation:**

A 28 year old woman, 37 weeks of pregnancy presented with fever of four days duration. She eventually developed irritability, altered sensorium, somnolence, and unresponsiveness to commands by the 5th day of febrile illness without any circulatory compromise. Physical examination and investigations including serology confirmed dengue fever. After excluding all other possible causes, the transient neurological deterioration was finally attributed to dengue encephalopathy which is an uncommon manifestation of the disease, particularly in pregnancy. Her deteriorated neurological status which had lasted for 6 days improved spontaneously with the convalescence of dengue infection. Cautious fluid management was carried out in correlation to clinical and hematological parameters. The pregnancy was continued uncomplicated till the platelet count had risen to more than 50,000 cells/cumm. She delivered vaginally a healthy male baby.

**Conclusions:**

Dengue fever in pregnancy is increasingly being encountered due to its rising disease burden. Dengue encephalitis/encephalopathy must be suspected in the differential diagnosis of fever and altered sensorium, even in pregnancy, in the tropical countries where the infection is rampant. Management of dengue infection in term pregnancy is a challenge for both the clinician and obstetrician. Further discussion and research are mandatory to decide on optimal management of these patients, with regard to monitoring, fluid management, and the precise timing and mode of delivery in order to prevent fatal morbidity and mortality to both mother and fetus.

## Background

Dengue is an mosquito-borne infection, caused by flavivirus serotypes (I–IV). It represents a significant burden of disease in the tropics, the global incidence is rising [1]. Dengue infection is a self limiting infection which may manifest with a spectrum of disease ranging from an uncomplicated febrile illness to various hemorrhagic manifestations and refractory shock. According to the world health organization dengue haemorrhagic fever is based on fever, thrombocytopenia and evidence of plasma leakage. Plasma leakage is confirmed in the presence of hemoconcentration (haematocrit rises 20% higher than the baseline) or any clinical evidence of bleeding.

Atypical manifestations in dengue are less recognized especially in a dengue epidemic situations [2]. Neurological involvement is one such example. Various neurological manifestations have been reported in literature [3]. These include encephalopathy, encephalitis, meningitis, myelitis, Guillain–Barre syndrome, myositis, optic neuritis to name a few. A typical neurological manifestations of dengue, particularly in pregnancy lacks a distinctive clinical picture and leads to diagnostic difficulties and poses a challenge to both the clinician and obstetrician. A high index of suspicion is mandatory in such clinical scenarios. We report a rare occurrence of a reversible dengue encephalopathy in a term mother with uneventful recovery.

## Case presentation

A 28 year old woman, 37 weeks of pregnancy presented with fever of four days duration to the casualty medical ward. The following day she appeared to be irritable, somnolent, aggressive and thereafter followed by unresponsiveness to commands. On examination she was febrile, anicteric and not pale. She was confused, poorly responsive and her GCS was 9 (eyeopening = 2, verbal response = 2, motor response = 5). Respiratory, cardiovascular and abdominal examinations were unremarkable. There was no neck stiffness, Kernigs and Brudzinki signs were negative. Cranial nerves were intact. Pyramidal and extrapyramidal signs were absent. Dilated fundoscopy did not reveal any papilledema. Her full blood count revealed an isolated thrombocytopenia. White cell count 9000 cells/cumm, Hemoglobin 10 g/dl, mean corpuscular volume 85 ft, Platelets 60,000 cells/mm, counts progressively dropping up until 8000 cells/cumm. Hematocrit was stable at 33%. ESR was 20 mm/h and was CRP 4 mg/dL. LDH was <200 u/L. Blood picture revealed a thrombocytopenia in keeping with a possible viral infection, there were no features of microangiopathic hemolytic anemia. Urine analysis excluded proteinuria and hematuria. Hepatic profile which was initially normal started to escalate. Aspartate aminotransferase 25 U/L up till 250 U/L (<40 U/L), alanine aminotransferase 20 U/L up till 250 U/L (<40 U/L), alkaline phosphatase 300 U/L (30–300 U/L) and serum bilirubin 0.7 mg/dL respectively, revealing an anicteric hepatitis which is a common finding in dengue infection. Renal functions and serum electrolytes were normal. Clotting profile was PT 10S, APTT 38S, INR 1.2 and FDP < 0.2 u/L, excluding the possibility of disseminated intravascular coagulation. Ultrasound scan did not reveal any pleural effusions or ascites excluding the early signs of capillary vascular leakage. Dengue non structural protein-1(NS) antigen was positive at day 4 of the illness. Dengue IgM antibodies were high in titre by day 6. Due to deterioration of clinical status she was transferred to intensive care unit for closer observation. An electroencephalography revealed bilateral rhythmic delta wave activity consistent with a diffuse cerebral dysfunction. CSF analysis was not performed due to profound thrombocytopenia, thus making it difficult for us to exclude other encephalitic pathogens as a cause for the encephalopathy. Nevertheless since serology was strongly positive for dengue infection and with the present dengue epidemic in our country we strongly suspected dengue encephalopathy as our first differential diagnosis. A CT scan of head was not performed due to possible risk of radiation to the fetus and the patients relatives refused consent for any imaging modalities. She was monitored closely, careful fluid management according to guidelines were carried out with regular fetal monitoring and bleeding precautions were undertaken. With the gradual rise in platelets, the patients neurological status improved spontaneously by day 10 of the illness. The encephalopathy had lasted approximately for 6 days. Fortunately, she did not have any clinical evidence of increased vascular permeability suggestive of dengue hemorrhagic fever or go into dengue shock syndrome. Once the platelet reached 70,000/mm^3^, she was induced for labour and delivered vaginally the baby without any peripartum or postpartum complications.

## Discussion

Neurological manifestations are uncommon in dengue infection. Encephalopathy is an unusual neurological complication of dengue fever. Its incidence ranges between 0.5 and 6%. Several studies report that DEN-2 and DEN-3 have the highest predisposition to neurological complications. The possible pathogenesis being described in literature include hepatic encephalopathy, cerebral hypoperfusion in dengue shock syndrome, cerebral edema due to increased vascular permeability, deranged electrolytes, and intracranial bleeding due to thrombocytopenia or coagulopathy [3–6]. The infection causes activation of cytokines and complements which result in endothelial dysfunction, platelet dysfunction and consumptive coagulopathy.

Dengue virus was primarily thought to be a non-neurotropic virus [7]. However, recent studies reveal the direct neuronal infiltration by the virus and the possible neurotropism leading to dengue encephalopathy and encephalitis. There are reports of demonstration of dengue virus and IgM antibody in the CSF of very few patients with encephalopathy/encephalitis [8, 9]. Virus or antibody can be isolated from the serum nevertheless CSF samples may be negative. In the case of our patient, we were unable to confirm the direct neuronal invasion with a CSF study due to profound thrombocytopenia, a strong limiting factor in confirming the diagnosis. Brain imaging modalities have shown a bilateral thalamic predisposition in dengue encephalitis [10]. However MRI findings in dengue fever have not been very clearly explained due to the paucity of the condition. Interestingly, Japanese encephalitis virus which closely resemble dengue virus characteristically produces bilateral thalamic involvement, hence being an important differential in the workup of the diagnosis [10] and can be confirmed by a CSF study. Though initially there was uncertainty about dengue encephalitis as an entity, it is established that dengue virus has neuroinvasive potential. The international encephalitis consortium has recognized this as an emerging area of encephalitis [11].

The diagnosis and management of dengue fever in pregnancy is a challenge to the clinician and the obstetrician. The physiological changes in pregnancy, pregnancy related complications and medical disorders must be kept in mind before interpreting the laboratory values. The entities in pregnancy that resemble dengue fever and need to be excluded include Idiopathic thrombocytopenic purpura (ITP) complicated with an intracranial haemorrhage, systemic lupus erythematosus (SLE) with cerebral vasculitis, antiphospholipid antibody syndrome (APLS) with CNS complications and pregnancy related complications such as pre-eclampsia, hemolysis, elevated liver enzymes and low platelets (HELLP), thrombotic thrombocytopenic purpura (TTP), sepsis and disseminated intravascular coagulation (DIC). The normal hematocrit levels in pregnancy may be as low as 33% (non pregnant female hematocrit is about 38%) [12]. As there is a physiological increase in the intravascular volume in the third trimester, the identification of plasma leakage syndrome through hemoconcentration or hypoproteinemia may be compromised which may under estimate the proportion of dengue haemorrhagic fever in pregnant women. There is increased risk of hemorrhage (maternal and fetal), pre eclampsia, miscarriages, fetal anomalies, low birth weight, premature births, increased rate of cesarean sections, increased maternal and fetal mortality and a risk of vertical transmission of dengue infection in pregnancy [13, 14].

Our patient is the first reported pregnant mother to suffer from dengue encephalopathy in the Indian subcontinent. Interestingly, the transient neurological deterioration spontaneously improved with the rise in platelet counts. This also raises the question of whether an immuno tolerant state such as pregnancy predisposes more to dengue encephalopathy/encephalitis.

In our patient, the suspicion of dengue encephalopathy was crucial for optimum monitoring and management to prevent adverse outcomes to both mother and fetus, although we were unable to confirm it with a CSF study. The current WHO guidelines do not elaborate on the clinical and hematological monitoring and fluid management of dengue infection in term pregnancy. More evidence based and multidisciplinary based studies are strongly and urgently indicated to decide on the diagnosis, value of CSF study, optimal fluid management with regard to dengue encephalopathy in a term pregnancy. The guidelines to determine the precise timing and mode of delivery for term mothers suffering dengue infection are essential to facilitate management and to prevent adverse outcomes.

## Conclusions

Dengue encephalopathy is a rare phenomenon complicating term pregnancy. This paper emphasizes on the hurdles of diagnosis and management of dengue fever in a term pregnancy, which definitely needs special attention. Further case reports and studies are mandatory to evaluate the exact magnitude and outcome of this clinical scenario as evidence-based data in the pathogenesis, diagnosis and the management of dengue encephalopathy in pregnancy is sparse.

## Abbreviations

ESR: erythrocyte sedimentation rate
CRP: C reactive protein
CT: computed tomography
GCS: Glasgow coma scale
LDH: lactate dehydrogenase
CSF: cerebrospinal fluid analysis
PT: prothrombin time
APTT: activated partial thromboplastin time
INR: international normalized ratio
FDP: fibrinogen degradation products
MRI: magnetic resonance imaging
